# A review of epidemiology of lymphatic filariasis in Nigeria

**DOI:** 10.11604/pamj.2024.47.142.39746

**Published:** 2024-03-27

**Authors:** Timothy Waje, Chanu Iliyasu, Lucy Musa Yaki, Ishaya Kato Auta

**Affiliations:** 1 Microbiology in Biological Sciences, Abubakar Tafawa Balewa University, P.M.B. 0248, Bauchi, Nigeria; 2 Biological Sciences, Nigerian Defense Academy, Kaduna, Nigeria; 3 Microbiology Department, Kaduna State University, P.M.B. 2335, Kaduna, Nigeria

**Keywords:** Lymphatic, filariasis, epidemiology, Nigeria, nematodes, microfilaria

## Abstract

Lymphatic filariasis is a neglected tropical disease that affects the lymphatic system of humans. The major etiologic agent is a nematode called Wuchereria bancrofti, but Brugia malayi and Brugia timoriare sometimes encountered as causative agents. Mosquitoes are the vectors while humans the definitive hosts respectively. The burden of the disease is heavier in Nigeria than in other endemic countries in Africa. This occurs with increasing morbidity and mortality at different locations within the country, the World Health Organization recommended treatments for lymphatic filariasis include the use of Albendazole (400mg) twice per year in co-endemic areas with loa loa, Ivermectin (200mcg/kg) in combination with Albendazole (400mg) in areas that are co-endemic with onchocerciasis, ivermectin (200mcg/kg) with diethylcarbamazine citrate (DEC) (6mg/kg) and albendazole (400mg) in areas without onchocerciasis. This paper covered a systematic review, meta-analysis, and scoping review on lymphatic filariasis in the respective geopolitical zones within the country. The literature used was obtained through online search engines including PubMed and Google Scholar with the heading “lymphatic filariasis in the name of the state”, Nigeria. This review revealed an overall prevalence of 11.18% with regional spread of Northwest (1.59%), North Central and North East, (4.52%), South West (1.26%), and South-South with South East (3.81%) prevalence. The disease has been successfully eliminated in Argungu local government areas (LGAs) of Kebbi State, Plateau, and Nasarawa States respectively. Most clinical manifestations (31.12%) include hydrocele, lymphedema, elephantiasis, hernia, and dermatitis. Night blood samples are appropriate for microfilaria investigation. Sustained MDAs, the right testing methods, early treatment of infected cases, and vector control are useful for the elimination of lymphatic filariasis for morbidity management and disability prevention in the country. Regional control strategies, improved quality monitoring of surveys and intervention programs with proper records of morbidity and disability requiring intervention are important approaches for the timely elimination of the disease in Nigeria.

## Introduction

Lymphatic filariasis, also called “elephantiasis” is a Neglected Tropical Disease (NTD) that affects and damages the lymphatic system of humans [1]. The major etiologic agent “*Wuchereria bancrofti*” is a microscopic thread-like worm responsible for about 90% of cases [2]. Other nematodes (roundworms) sometimes encountered in the etiology of the disease are *Brugia malayi and Brugia timori* [1]. These worms thrive well in tropical climates where mosquitoes which are their vectors are found [3]. The parasites are transmitted through mosquito bites [4]. In Africa, *Anopheles* mosquitoes are the common vectors, *Culex quinquefasciatus* in America, while *Aedes* and *Mansonia* in the Pacific and Asia [5]. The various vector species involved in the disease transmission include: i) Anopheles: *Anopheles arabinensis, Anopheles bancrofti, Anopheles gambiae, Anopheles melas, Anopheles punculatus, Anopheles farauti, Anopheles merus, Anopheles wellcomei* among others. ii) Culex: *Culex quinquefasciatus, Culex pipiens, Culex annulirostris, Culex bitaeniorhynchus*. iii) Aedes: *Aedes bellator, Aedes aegypti, Aedes cooki, Aedes rotumae, Aedes darlingi, Aedes kochi, Aedes vigilax, Aedes scapularis*, and *Aedes polynesiensi*. iv) Mansonia: *Mansonia uniformis*, and *Mansonia pseudotitillans* [2].

*Wuchereria bancrofti* exhibits sexual dimorphism (a condition where a male and female of the same species exhibit different characteristics in addition to sex organs), always in pairs with the male having a curved tail, additional sensory organs, about 40mm in length, 100 micrometers in width and smaller than the female which is approximately 60mm in length and 100mm in width with a tapered rounded tip without sensory organs [5,6]. There is a pair of dissimilar penial setae or copulatory spicules in the cloacal or curved regions and many copulatory papillae in the posterior end [7]. The male and female are always found coiled together [6]. The adult worm is white to transparent, fragile, elongated, and cylindrical [8].

**Figure 1:**
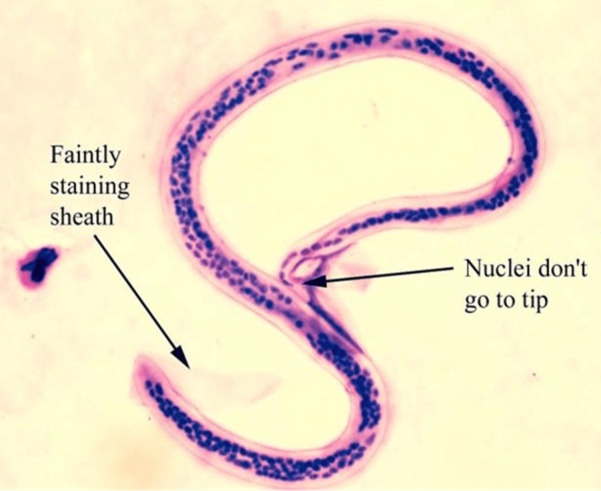
Wuchereria *bancrofti* microfilaria

The life cycle of *Wuchereria bancrofti* involves two hosts, namely: humans, the definitive hosts, and mosquitoes, intermediate hosts, respectively [5]. It begins with the introduction of a third larval stage to humans during mosquito blood meal [2]. The larva spreads to the lymphatic system via blood circulation and develops into maturity in about 6 to 9 months [9]. These adult worms reside in the lymphatic system and produce sheathed microfilariae, which are found in the blood circulation and lymphatics of their hosts [2]. The third stage larva is actively motile and is the infective stage which can be introduced to humans via blood meal by mosquitoes [5]. The microfilariae exhibit nocturnal periodicity by staying in the deep blood vessels of the definitive host during the day but migrating to the surface of peripheral or superficial blood vessels at night [5]. Mosquitoes ingest microfilariae from infected humans, which then migrate through the walls of their proventriculus and the cardiac region of the midgut to the thoracic muscles where they develop into a first-stage larva, second-stage and third-stage larva respectively (Figure 1) [2,10].

Filariasis is the disease caused by *Wuchereria bancrofti* [2]. The filarial worms reside in the lymphatic pathways of humans, obstructing the flow of lymph and causing a condition called elephantiasis or lymphatic filariasis [8]. Lymphatic filariasis of *Wuchereria bancroftian* etiology is also called bancroftian filariasis [7]. Although the disease can be asymptomatic for a long time, this asymptomatic infection damages the kidneys, lymphatic system, and the immune system gradually [1]. A few individuals develop lymphedema; fluid collection is due to dysfunction of the lymphatic system resulting in swelling of the legs, arms, breasts, and genitals after prolonged infection [2]. There is fever, chills, eosinophilia (presence of higher-than-normal white blood cells), granulomatous lesions, lymphangitis, lymphadenitis, epididymis orchitis, and lymphadenopathy [5,7]. Further complications lead to bulky and lumpy with stiff tough skin, pain, and general body malaise [4]. There is hydrocele (involving the scrotum), chyluria/milk in urine, hematuria/blood in urine, or proteinuria/protein in urine (renal involvement) [9]. Lymphatic filariasis can be diagnosed from blood samples taken at night (due to microfilariae nocturnal periodicity) through microscopic investigation of microfilariae in blood smear, immunoassay for IgG4, polymerase chain reaction (PCR) for parasite DNA or Immunochromatographic card test (ICT) for detection of circulating filarial antigen (CFA) [5].

The World Health Organization recommended treatments for lymphatic filariasis include the use of: Albendazole (400mg) twice per year in co-endemic areas with loa loa, ivermectin (200mcg/kg) combined with albendazole (400mg) in areas co-endemic with onchocerciasis, ivermectin (200mcg/kg) with diethylcarbamazine citrate (DEC) (6mg/kg) and albendazole (400mg) together safely clear the microfilariae among infected persons in areas without onchocerciasis within few weeks [1,5]. Diethylcarbamazine citrate kills the microfilariae but has side effects such as pain in joints, fever, nausea, vomiting, dizziness, and worsening onchocercal eye disease in co-endemic areas with onchocerciasis [2]. The microfilariacidal and nematocidal actions (higher doses required for adult worms) of these drugs include: albendazole (400mg) - which disrupts the worm microtubule of the cytoskeleton, ivermectin (200mcg/kg), disrupts glutamate-gated chloride channels which control the release of secretory vesicles that interfere with the host´s immune response and DEC (6mg/kg) targets the arachidonic metabolic pathways (eicosanoids biosynthesis) against microfilariae sensitizing them for phagocytosis [5]. Surgery is important for those with hydrocele [7]. Vector control, which involves the use of mosquito nets, insecticides, and repellants, is an important preventive measure for the transmission of *Wuchereria bancrofti* [1]. Treatment of infected cases can prevent the spread of the causative agents [3].

Epidemiology involves the study of the distribution (frequency and pattern) and determinants in relation to causes and risk factors for health-related issues and diseases in a specified population with application for prevention and control [11]. Lymphatic filariasis is a widely distributed disease because the etiologic agent “*Wuchereria bancrofti* is ubiquitous in the tropics and subtropics, common in Central and West Africa, Nile Delta, Thailand, Pakistan, India, Korea, Japan, Philippines, in a geospatial analysis report, an estimated global population of 199 million people was infected with lymphatic filariasis, including 3.1 million people in America and 107 million in South East Asia by 2000 but experienced a sharp decline by 2018 except in Africa and South East Asia where local elimination are yet to reach the threshold [12]. Furthermore, lymphatic filariasis has been reported endemic in sub-Saharan Africa (except the Southern region of the continent), Madagascar, and many nations in the Western Pacific Island and Territories as well as parts of the Caribbean [13]. The distribution of the disease has been described as heterogeneous with affinity to certain geographical locations and requires sustained treatment for elimination [14]. In North Africa, the transmission of lymphatic filariasis in Egypt has been interrupted by successful mass drug administration (MDA), achieving a prevalence rate of less than 1%, which meets the WHO standard for successful elimination [15]. The WHO MDA records of 2021 declared Algeria, Libya, Morocco, and Tunisia non-endemic [16].

In East Africa, a prevalence rate of 1.60% was reported in South Sudan and associated with high poverty, low literacy level, household clustering, and poor vector control [17]. Similarly, 5.80% was reported for CFA in 15 communities of the Mkinga district and Tanga region in Tanzania and attributed to exposure to vectors and low MDA coverage [18]. Furthermore, 5.51% of CFA with lymphedema and hydrocele was reported in the Tanga region, North Eastern Tanzania, and attributed to the postponement of MDA [19]. Yumbe, Kitgum, and Lira regions of Uganda were considered endemic to Lymphatic filariasis and associated with border proximity to South Sudan, weakened health and social support systems, inadequate awareness as well as low MDA coverage [20]. The World Health Organization on MDA status classified Burundi and Rwanda, Seychelles, Somalia, and Mauritius as non-endemic but Kenya, Ethiopia, Madagascar, Mozambique, Zambia, Eritrea, Comoros, Zimbabwe, and Democratic Republic of Congo (DRC) endemic with ongoing MDA while Uganda under surveillance but successful elimination in Malawi [15]. Similarly, in the Central African sub-region, endemic countries with ongoing MDA include: the Central African Republic (CAR), Chad, and Cameroon but MDA has not commenced in Equatorial Guinea, Gabon, and Sao Tome and Principe among others [21]. In West Africa, Gambia, Mauritania, and Cape Verde are classified as non-endemic but elimination is successful in Togo while Benin Republic, Liberia, Ghana, Côte d´Ivoire, Guinea, Guinea Bissau, Senegal, Sierra Leone, Niger, Burkina Faso and Nigeria are endemic undergoing MDA while Mali under surveillance [15] (Table 1).

**Table 1:** endemicity and MDA status of lymphatic filariasis in some African countries as of 2021

African sub-region	Countries	Endemicity	Prevalence rate (%)	MDA status
North Africa	Egypt	Eliminated	≤1	Successful elimination
	South Sudan	Endemic	1.60	Ongoing
	Algeria, Libya, Morocco Tunisia	Non-endemic	-	No MDA
East Africa	Ethiopia, Madagascar, Kenya, DRC, Eretria Mozambique, Zambia, Zimbabwe, Comoros	Endemic	-	Ongoing
	Tanzania	Endemic	5.80, 5.51	Ongoing
	Malawi	Eliminated	≤1	Successful elimination
	Uganda	Surveillance	None	Under surveillance
	Burundi, Rwanda Somalia, Mauritius, Seychelles	Non-endemic	-	-
Central Africa	CAR, Chad, Cameroun	Endemic	-	Ongoing
	Equatorial Guinea, Gabon, Sao Tome and Principe	Endemic	-	-
West Africa	Gambia, Cape Verde, Mauritania	Non-endemic	-	-
	Benin, Niger, Liberia, Ghana, Cote d'Ivoire, Guinea, Senegal, Burkina Faso, Guinea Bissau, Sierra Leon, Nigeria	endemic	-	Ongoing
	Mali	Surveillance	-	Under surveillance

MDA: mass drug administration

Neglected tropical diseases elimination programs for lymphatic filariasis decrease transmission and infection rates in endemic areas [2]. This is achieved through the annual treatment of whole communities through MDAs (preventive chemotherapy with safe medication), basic care for those with complications, and avoidance of mosquito bites [22,23]. The global burden of vector-borne diseases is considered significant and as such community mobilization and vector control are important preventive methods [24]. Therefore, this review is aimed at identifying the research and control gaps associated with lymphatic filariasis in Nigeria in the context of Africa to spur researchers, intervention organizations, and the Government to eradicate the parasite for public health improvement. The specific objective of this paper is to identify endemic locations within Nigeria, the risk of transmission of the causative agents, disease burden/reported symptoms, effective diagnostic methods, the spread of the parasite (prevalence) as well as interventional status for the elimination of the disease. A few questions of concern include: i) What are the contributors to the lymphatic filariasis disease burden in Nigeria? ii) Why has the eradication of the disease taken so long despite intervention programs? iii) What strategy is required for an effective and timely eradication of lymphatic filariasis in the location?

## Methods

**Study type and location:** this paper involves a systematic review, meta-analysis, and scoping review of lymphatic filariasis within the respective geopolitical zones in Nigeria. Literature searches were made in PubMed, Scopus, and Google Scholar using the heading “lymphatic filariasis” in the named State within the country [25].

**Inclusion and exclusion criteria:** all useful publications were sorted out while unrelated ones and duplicates were discarded. The inclusion criteria were published articles on lymphatic filariasis or *Wuchereria bancrofti*, articles of original research, reviews, and research carried out in Nigeria within the 6 geo-political zones respectively. Articles with reports that included a population of study, type of test carried out, outcome of research, and possible risk of acquisition of the disease were considered eligible while those without clear and sufficient or irrelevant reports were discarded.

**Figure 2:**
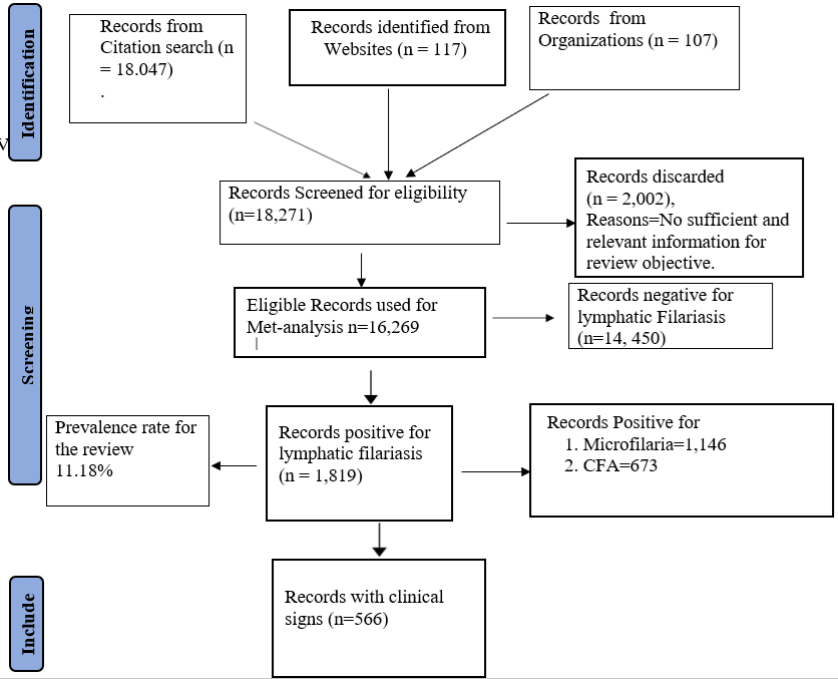
PRISMA flow chart for review of the epidemiology of lymphatic filariasis in Nigeria

**Sample size estimation and data extraction:** the sample size was determined by the summation of records (population sizes) in eligible reports at the various regions within the country. Data was extracted from the respective methodologies (type of test such as rapid diagnostic test (RDT) for CFA or microscopy of thick blood smear for identifying parasitemia), test results/findings (positive or negative), report for presence and absence of clinical signs, and risks for transmission in discussions of these reports. All data extrapolated were verified and approved by co-authors for further processing [26]. The Preferred Reporting Items for Systematic Review and Meta-analyses (PRISMA) flowchart in Figure 2 summarizes the data synthesis from eligible reports [27].

**Review scope and prevalence rate determination:** the review focused more on qualitative than quantitative analysis according to the study objective. The overall prevalence rate of the disease in the country was determined using the formula for prevalence rate determination.

Therefore, endemic locations (prevalence rate) within Nigeria, risk of transmission of the causative agents, disease burden/reported symptoms, diagnostic methods, and interventional status for elimination of the disease were identified and tabulated respectively.

## Results

**Figure 3:**
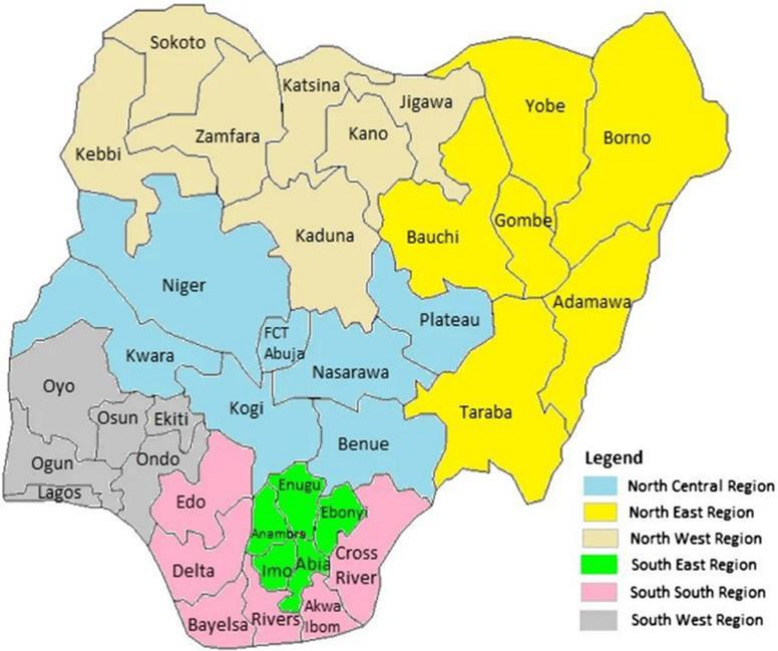
map of Nigeria with 36 States and six geopolitical zones

Nigeria has 36 States and a federal capital territory (FCT) which are spread across six geopolitical zones as North West, North East, North Central, South West, South East, and South-South respectively [28,29] (Figure 3). Out of the 18,271 records which correspond to the total population size of the different reports screened for this review´s eligibility, 16,269 were eligible. The remaining 2,002 ineligible records were discarded. The total number of the population reported positive for lymphatic filariasis is 1,819 with a prevalence rate of 11.18% out of which 1,146 (63.00%) were positive for microfilaria and 673 (37.00%) for CFA. The remaining 14,450 were reported negative for both microfilaria and CFA. The geopolitical zones that make up for the national prevalence rate of 11.18% include the Northwest 1.59%, North Central with the North East 4.52%, South West 1.26%, and South-South with South East 3.81% respectively. Among the positive subjects in the various records, 566 (31.12%) were reported with different clinical symptoms. This result and the geopolitical zone prevalence are summarized in Table 2.

**Table 2:** prevalence of lymphatic filariasis in Nigeria

Geo- political Zones	Eligible records	Lymphatic filariasis				Presence of microfilaria		Presence of CFA		Positive no with symptoms	
	Population size	No positive for CFA and microfilaria		Percentage (%) of eligible records	Regional prevalence rate (%)	No positive	Percentage (%) of total positive records	No positive	Prevalence rate (%)	No with symptoms	Percentage of positive population (%)
NW	6,890	258	1.59		3.74	8	0.44	250	13.74	82	4.51
NC & NE	3,859	736	4.52		19.07	563	30.95	173	9.51	435	23.91
SW	2,251	205	1.26		9.11	123	6.76	82	4.51	3	0.17
SS & SE	3,269	620	3.81		18.97	452	24.85	168	9.24	46	2.53
Total	16,269	1,819	11.18		50.89	1,146	63.00	673	37.00	566	31.12

NW=North West, NC-North Central, SW=South West, SS=South-South, SE=South East, CFA=circulating filarial antigen

**Lymphatic filariasis in North West Nigeria:** in North West Nigeria, a micro-stratification overlaps mapping (MOM) of lymphatic filariasis data from literature and surveillance reported a 10.00% CFA prevalence and 0.3% microfilaria for Kaduna [30]. Similarly, 7.30% of CFA through ICT and 0.90% through microscopy were reported for Katsina State by mapping the baseline prevalence outcome [31]. Furthermore, a seroprevalence rate of 37.80% CFA by rapid diagnostic test (RDT) with poor sanitation, proximity to water bodies, and lack of awareness is reported in a study of six communities of Talata Mafara Local Government Area of Zamfara State, Nigeria [32]. A CFA prevalence rate of 1.10% by ICT and microfilaria of 1.6% (using the thick blood film method) with symptoms of hydrocele, adenolymphagitis, elephantiasis, and hosts´ exposure to vectors was reported in a study in three LGAs of Kano State [33]. A baseline mapping study reported a CFA prevalence of 6.8% in Jigawa State [31]. Similarly, a 2.00% microfilariae prevalence through microscopy of thick film Giemsa-stained blood smears with 11.30% lymphedema and 7.00% hydrocele were reported in three communities of Jahun Local Government Area in Jigawa State [34]. In Sokoto State, a 10.00% prevalence of CFA through combo rapid test kits with hosts´ exposure to vectors was reported in five wards in Bodinga Local Government Area [35]. A low prevalence of 0.40% was reported through ICT in 6 rural communities of Argungu LGA in Kebbi State meeting WHO criteria of less than 1.00% positivity for endemicity /successful elimination) [36] Table 3.

**Table 3:** lymphatic filariasis in Northwest Nigeria

S/N	States	Study coverage/local government area (LGAs)	Test type	Micro-filariae prevalence rate (%)	Circulating filarial antigen prevalence (%)	Reported symptoms	Possible associated risks
	Kaduna	State	Microscopy/ICT	0.30	10.00	-	-
	Zamfara	6 communities in Talata Mafara LGA	RDT	-	37.80	-	Poor sanitation, proximity to water bodies, and lack of awareness
	Kano	3 LGAs	Thick blue film microscopy/ICT	1.60	1.10	Hydrocele, adenolymphagitis limp elephantiasis	Exposure to vectors
	Jigawa State	3 communities of Jahun LGA	Microscopy/ICT	2.00	6.80	Lymphedema and hydrocele	-
	Katsina	State	ICT/data mapping	0.90	7.30	-	-
	Sokoto	5 wards in Bodinga LGA	RDT	-	10.00	Nil	Exposure to vector
	Kebbi	6 rural communities in Argungu LGA	ICT/microscopy	0.00	0.40	Eliminated	Successful elimination

ICT: immunochromatographic card test, RDT: rapid diagnostic test

**Lymphatic filariasis in North Central and North East, Nigeria:** a high endemicity (32.60%) of CFA prevalence through ICT with 8.50% hydrocele, 6.40% lymphedema, and subjects´ need for awareness of the disease was reported in Ado LGA of Benue State [37]. Although Kogi State is reported with the highest number of people living with NTDs in Africa, 3.40% microfilaria prevalence from blood samples collected at night (between 9.00 PM-12.00 AM) with dermatitis (3.70%), hydrocele (0.70%), elephantiasis (3.70%), and hosts´ exposure to vectors were reported among 5 communities in Yagba West LGA of the State [38,39]. On the other hand, a successful elimination of the parasite in the Plateau and Nasarawa States respectively was reported in 2017 [40]. In a comparative study between diagnostic techniques in the same population, 26.19% prevalence for microfilaria through thick film microscopy and 31.29% for CFA through ICT with suggestive poor vector control and sanitation were reported in Northern Taraba State [41]. Similarly, a 33.84% prevalence rate was obtained through microscopy of thick film Giemsa-stained blood smear from blood samples collected at night (between 8.00 PM-01.00 AM) with symptoms of itching, hydrocele, lymphedema, elephantiasis, adenolymphagitis were reported for muri emirate (11 LGAs) in Taraba State [42]. Furthermore, 1.50% CFA prevalence through Filarial Test Strips (FTS) with hydrocele and inadequate awareness of the disease were reported among eleven communities in eleven LGAs of Borno State [43]. Lymphatic filariasis was reported in Galdamaru Kaltungo LGA of Gombe State in 2018 [44]. A microfilariae prevalence of 35% among villagers of Biliri and Balanga LGAs with the need for awareness on mosquito vector control was reported in the State [45] (Table 4).

**Table 4:** lymphatic filariasis in North Central and North East Nigeria

S/N	States	Study coverage/local government area (LGAs)	Test type	Micro-filarial prevalence rate (%)	Circulating filarial antigen prevalence rate	Reported symptoms	Possible associated risks
	Benue	Ado	ICT	-	32.60	Hydrocele and lymphedema	Inadequate awareness
	Kogi	Yagba LGA	Microscopy	-	3.40	Dermatitis, hydrocele, and elephantiasis	Exposure to vectors
	Plateau	-	-	≤1	-	Eliminated	Successful elimination
	Nasarawa	-	-	≤1	-	Eliminated	Successful elimination-
	Taraba	Eleven communities of northern Taraba	Thick film giemsa-stained smear microscopy/ICT	26.19	31.29	-	Poor vector control and sanitation
	Taraba	Seven communities in Muri emirate of Jalingo LGA	Thick film giemsa-stained smear microscopy	33.84	-	itching, hernia hydrocele, lymphedema, elephantiasis adenolymphagitis	Exposure to vector, and rural settlement
	Borno	Eleven communities in 11 LGAs	ICT/FTS	-	1.50	Hydrocele	Inadequate awareness
	Gombe	Galdamaru, Kaltungo LGA	-	-		Elephantiasis	Boarder with endemic areas
		Biliri and Balanga	Thick blood giemsa-stained microscopy	35.00	-	-	Inadequate awareness and vector control

ICT: immunochromatographic card test, FTS: filarial test strips

**Lymphatic filariasis in South West Nigeria:** a microfilaria prevalence rates of 4.00% and 2.40% by microscopy of Giemsa-stained night blood samples (collected between 10.00 PM-02.00 AM) with inadequate awareness of the cause of lymphatic filariasis were reported for Ado-Odo Ota and Abeokuta South LGAs of Ogun State [46]. Similarly, 17.00% microfilaria prevalence with 2.20% hydrocele and elephantiasis as well as vegetation, lack of drainages, and presence of vectors such as Aedes, *Culex*, and *Anopheles* mosquitoes were reported in Sowo Village in Abeokuta [47]. Furthermore, a prevalence rate of 20.30% for microfilaria through microscopy of Giemsa-stained thick blood films from night blood samples with inadequate awareness of transmission, treatment, and prevention of the disease was reported among six communities in Imobi, Ijebu East LGA of Ogun State [48]. An immunochromatographic card test revealed a CFA prevalence of 1.70% with a case of hydrocele and some *Anopheles. Gambiae* infected with *Wuchereria bancrofti* in 10 selected communities of 5 LGAs in Osun State [49]. A prevalence rate of 29.00% for CFA through ICT with few cases of morbid symptoms such as 0.43% hydrocele and 0.43% lymphedema as well as risks for spread such as inadequate knowledge of the disease and poor vector control were reported in three rural communities (Idoani Imeri, and Idogun) in Ose LGA of Ondo State [50] (Table 5).

**Table 5:** lymphatic filariasis in some locations of South Western Nigeria

S/N	States	Study coverage/local government area (LGAs)	Test type	Micro-filariae prevalence rate (%)	Circulating filarial antigen prevalence (%)	Reported symptoms	Possible associated risks
	Ogun	Ado-Odo, Otta, LGA	Giemsa-stained smear microscopy	4.00	-	-	Lack of awareness and vector control
	Ogun	Abeokuta South LGA	Giemsa-stained smear microscopy	2.40	-	-	Lack of awareness and vector control
	Ogun	Sowo village, Abeokuta	Giemsa-stained smear microscopy	17.00	-	Elephantiasis hydrocele, dermatitis	Vegetation, lack of drainages and presence of vectors
	Ogun	6 communities in Imobi, Ijebu East LGA	Giemsa-stained smear microscopy	20.30	-	-	Lack of awareness, exposure to vectors
	Osun	10 communities in 5 LGAs	ICT	-	1.70	Hydrocele	vegetation, exposure to vectors
	Ondo	3 rural communities of Ose LGA	ICT	-	29.00	Lymphedema and hydrocele	Lack of awareness and poor vector control

ICT: immunochromatographic card test

**Lymphatic filariasis in South-East, and South-South Nigeria:** a microfilariae prevalence of 24.33% obtained through microscopy of Giemsa-stained thick blood smear from night samples (collected 10.00 PM-12.00 AM) was reported in three LGAs (Owerri North, Owerri West, and Ngor Okpala LGAs) of Imo State [51]. In Igbo-Eze North LGA of Enugu State, a high prevalence rate of 41.80% obtained through microscopy of Giemsa-stained thick film blood smear with associated risks of poor vector control, proximity to water bodies and lack of knowledge/awareness was reported in 4 communities [52]. Similarly, in Ebonyi State, prevalence rates of 5.38% microfilaria and 21.13% CFA with 8.23% hydrocele and 8.88% lymphedema and contraction risks of exposure to vectors and poverty were reported among inhabitants of 30 communities of Afikpo North LGA [53]. Furthermore, 22.30% was reported using standard parasitological techniques (thick film smear) with farming and fishing as risks of contracting the disease among eight communities of Ukwa East Local Government Area of Abia State [54]. An overall microfilaria prevalence of 20.20% from standard parasitological methods with exposure to vectors, presence of stagnant water, and farming as risks of contraction of the disease were reported among inhabitants of Ogidi in Idemili North LGA of Anambra State [55]. Similarly, in Yakurr LGA, a microfilaria prevalence of 6.10% through thick blood smear microscopy with lymphedema (0.30%) and exposure of residents to vectors were reported in 4 communities [56]. A CFA prevalence of 1.00% with inadequate knowledge of the disease and exposure of residents to vectors were reported among residents of 4 communities in Yenagoa LGA of Bayelsa State [57] (Table 6).

**Table 6:** lymphatic filariasis in some parts of South East and South-South Nigeria

S/N	States	Coverage/local government area (LGAs)	Test type	Micro-filariae prevalence rate (%)	Circulating filarial antigen prevalence (%)	Reported symptoms	Associated risk factor
	Imo	3 Communities in 3 LGAs	Giemsa-stained thick blood films microscopy	24.33	-	-	Poor vector control
	Enugu	Igbo-Eze North LGA	Giemsa-stained Smear microscopy	41.80	-	Lymphedema	Exposure to vectors, proximity to water bodies. lack of awareness.
	Ebonyi	30 communities in Afikpo North LGA	Giemsa-stained thick blood films microscopy/ICT	5.38	21.13	Hydrocele and lymphedema	Exposure to vectors and poverty
	Abia	8 communities (Ndoki people) of Ukwa East LGA	Thick blood films smear microscopy	22.30	-	Lymphedema	Farming and fishing-
	Anambra	Ogidi community of Idemili North LGA	Thick film giemsa- stained smear microscopy	20.20	-	-	Poor vector control, stagnant water and farming
	Cross River	3 communities of Yakurr LGA	Thick film giemsa- stained smear microscopy	6.10	-	lymphedema	Exposure to vectors, and poor vector control
	Cross river	Yakurr LGA	Blood smear microscopy	6.10		Lymphedema	Exposure to vectors and poor sanitation
	Bayelsa	4 communities, Yenagoa LGA	ICT	-	1.00	-	Inadequate knowledge exposure vector

ICT: immunochromatographic card test

It is important to note that in each geographical location, positive cases of lymphatic filariasis have been reported at one location or the other and this review is inexhaustible as there could be other locations not captured. Furthermore, border proximity to endemic States is also a risk for the spread of this disease [20].

## Discussion

The epidemiology of lymphatic filariasis, like of other NTDs has been described as complex and associated with environmental conditions in tropical areas [58]. Other NTDs are Buruli ulcer, Chagas disease, cysticercosis, dengue fever, echinococcosis, fascioliasis, trypanosomiasis, dracunculiasis (Guinea Worm disease), leishmaniasis, leprosy, onchocerciasis, schistosomiasis, trachoma, soil-transmitted helminths (STHs, Ascaris, Hookworm, and Whipworm) among others [59]. They constitute a diverse set of over 20 diseases with debilitating symptoms and devastating health, economic, and social effects on a large population of the world [58]. Nigeria is reported with the highest burden of lymphatic filariasis compared to other endemic counties in Africa [31].

The overall prevalence of 11.18% obtained in this review with the occurrence of various clinical manifestations (31.12%) such as hydrocele, lymphedema, dermatitis, hernia, itching, and breast enlargement among positive persons suggest transmission is still ongoing. This suggests a need for more interventions to meet WHO criteria of less than 1.00% positivity for a successful elimination which agrees with a previous report on the high burden of lymphatic filariasis in Nigeria [25,36]. This could probably be attributed to poor vector control and rural-urban migration despite interventions. Similarly, the country was reported with 14.30% of the global population requiring intervention through preventive chemotherapy as of 2017 [30]. However, transmission suitability varies across the country due to differences in environmental and climatic factors favoring vectors with the highest mean seroprevalence predicted for North Central (8.2%), North West (7.8%), and South East (7.1%) while South-South with 2.5% prevalence [32]. The geopolitical zones prevalence from the 11.18% obtained in this review such as Northwest (1.59%), North Central with the North East (4.52%) South West (1.26%), and South-South with South East (3.81%) respectively revealed almost the same trend. The reduction in the respective prevalence rates could be attributed to the impact of interventions. Nevertheless, regional studies are necessary for the diversification of control strategies.

In Kano and Jigawa States of Northwest Nigeria, hydrocele, adenolymphagitis, lymphedema, and elephantiasis have been reported among some positive individuals while Zamfara, Sokoto, Kaduna, and Katsina States had no reported symptoms of the disease but a successful elimination in some parts of Kebbi State [33-36]. Major risks for the spread of the etiologic agent in this region include poor sanitation and vector control, as well as inadequate awareness among the inhabitants. Therefore, regional strategy for vector control, parasite elimination, prevention of spread by migrants, and elimination in communities bordering endemic areas could be of great value to existing elimination programs in the North West of Nigeria.

In the North East, and North Central of Nigeria, the disease is reported eliminated in Plateau and Nasarawa States, but hydrocele, lymphedema, fever, elephantiasis, hernia, dermatitis, and itching among positive persons with poor sanitation, inadequate awareness, exposure to vector and border proximity to endemic areas as risks factors were reported in Benue, Kogi, Taraba, Gombe and Borno States respectively [38-41]. Nevertheless, a mechanism of independent assessment for the quality of intervention and a post-survey to ensure the integrity of data from pre-transmission assessment survey (pre-TAS) or transmission assessment survey 1 (TAS1) could improve the quality of intervention programs. Furthermore, awareness creation in endemic and non-endemic communities as well as enforcement of environmental control processes such as clearing of vegetation, improving drainages, and proper refuge disposal which destroy breeding sites and dispel vectors from the environment could help in reducing the duration of intervention in the North East and North Central respectively. In southwest Nigeria, dermatitis, hydrocele, lymphedema, and elephantiasis as clinical manifestations with exposure to vectors, poor drainage, vegetation, and inadequate awareness as risks of the disease spread have been reported among positive persons in some parts of Osun, Ogun, and Ondo States respectively [46-50].

A regional strategy in the South West which includes: survey and intervention coverage expansion, effective rural-urban migration screening and control for infected persons, and adequate campaigns, as well as more involvement of government and intervention organizations, could improve the quality and duration of interventions in the region. In the South East, and South-South Nigeria, dermatitis, hydrocele, lymphedema, and elephantiasis have been reported among some positive persons with the exposure of residents to vectors, favorable breeding sites (stagnant water, vegetation), poor sanitation for vectors, occupation (farming and fishing), poor health-seeking behavior, and rural-urban migration have been reported in Imo, Enugu, Ebonyi, Abia, Cross River, and Edo States respectively [51-56]. The peculiarity of the region requires a unique approach to the elimination of the disease through regional concentration on morbidity management and disability prevention, proper records for persons with complications (morbidity and disability) and tracking of interventions and impact on casualties, risk mitigation strategy for persons in certain occupations such as farming and fishing. Although this review relied on literature without sight of reviewed locations, it was slowed by internet network fluctuations and had a risk of transferred errors from sources. Nevertheless, it provides information on interventional coverage, disease burden, risks of spread, and effective laboratory testing methods which are useful to Government and interventional organizations, policymakers, and researchers for the successful elimination of lymphatic filariasis in Nigeria.

## Conclusion

The burden of lymphatic filariasis is heavy in Nigeria with cases of elephantiasis, hydrocele, dermatitis, lymphedema, and hernia, this is of public health concern. Proximity to stagnant water, vegetation, poor vector control, rural-urban migration, and inadequate awareness have been potential risks for the spread of the parasite within the country. Elimination programs have been useful in decreasing morbidity, mortality, and transmission rates with the improvement of public health in endemic areas of the world and the nation at large. Therefore, successful morbidity management and disability prevention involve control strategies to include: reliable test type, night blood sampling and the use of ICT or thick blood smear giemsa-stained microscopy, simultaneous preventive chemotherapy, and vector control. Nevertheless, regional strategies, quality control checks, integrity of data, sustained interventions, maintaining records of persons with disability and complications, and control of transmission through migrants are effective and timely control methods. Although positive cases have been reported at some locations of each geographical zone, the risk of spread across borders due to proximity to endemic areas and interstate movements is of concern. On this note, intervention organizations, relevant government agencies, and public health professionals should intensify campaigns for awareness, vector control through the use of insecticides, nets, repellants, and destruction of breeding sites as well as early treatment of infected cases as good preventive measures and reduction of transmission or complication development. Therefore, the environment, vector, and hosts in the respective geo-political zones are all to be considered for the successful elimination of lymphatic filariasis in Nigeria.

### What is known about this topic

- *The burden of lymphatic filariasis is heavy in Nigeria with complications such as elephantiasis, lymphedema, dermatitis, hernia, and hydrocele;*
- *Proximity to stagnant water, vegetation, exposure to vectors, rural-urban migration, and inadequate awareness contribute to the spread of the disease;*
- *Due to microfilariae nocturnal periodicity, reliable test result is achieved by testing blood samples collected at night*.

### What this study adds

- *Elimination strategy in communities bordering endemic areas is of great value to existing intervention programs in Nigeria;*
- *Enforcement of environmental control programs such as clearing of vegetation, improving drainage, and proper refuge disposal which destroy breeding sites and dispel vectors as important vector control tools;*
- *Rural-urban migration screening and control for infected persons especially from endemic areas, documentation and tracking of interventions for persons with complications, and risk mitigation strategies for persons in exposed occupations (fishing and farming) are important for the timely elimination of the diseases*.
